# High-resolution spatiotemporal mapping: a comprehensive view of eukaryotic cell cycle proteome dynamics

**DOI:** 10.1038/s41392-024-01850-z

**Published:** 2024-05-22

**Authors:** Jing Zou, Ziran Qin, Long Zhang

**Affiliations:** 1 Life Sciences Institute, Second Affiliated Hospital of the Zhejiang University School of Medicine, The MOE Key Laboratory of Biosystems Homeostasis & Protection and Zhejiang Provincial Key Laboratory for Cancer Molecular Cell Biology, Zhejiang University, Hangzhou, Zhejiang, China; 2 The MOE Basic Research and Innovation Center for the Targeted Therapeutics of Solid Tumors, The First Affiliated Hospital, Jiangxi Medical College, Nanchang University, Nanchang, China; 3 Cancer Center, Zhejiang University, Hangzhou, Zhejiang, China

**Keywords:** Imaging, Molecular biology

In a recent study published in *Cell*, Litsios et al. revealed a high-resolution spatiotemporal map of the eukaryotic cell cycle proteome.^1^ They identified proteome-level changes in both abundance and spatial distribution throughout the cell cycle and provided a valuable resource for future exploration into the global proteome dynamics that drive cell cycle progression (https://thecellvision.org/cellcycle).

**Fig. 1:**
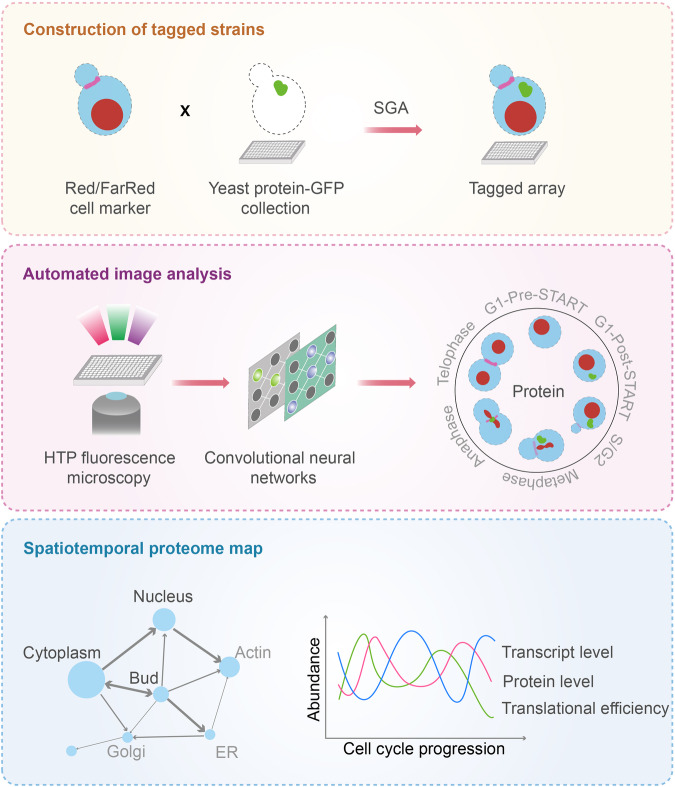
Schematic overview of the high-content screening workflow for mapping spatiotemporal proteome dynamics. Tagged strains were constructed using the synthetic genetic array (SGA) method to introduce extra red and far-red fluorescent markers into every strain in the GFP collection (the nucleus and bud neck in red/pink, cytoplasm in blue, and the protein of interest in green). High-throughput (HTP) fluorescence microscopy was employed to capture images of budding yeast cells. These cells were automatically segmented into single cells and then classified into specific cell cycle stages and subcellular localization using convolutional neural networks. Mapping the landscape of proteome movements (left panel) and integrating multi-omics data (right panel) to unveil a comprehensive genome-scale spatiotemporal map of the yeast cell cycle proteome. In the left panel, the size of the dots represents the number of protein changes observed at the indicated localizations, and the thickness and direction of the arrows signify the direction and magnitude of protein movement. In the right panel, three curves representing the periodic changes of the protein of interest across the cell cycle at the levels of transcription, translation efficiency, and protein abundance, respectively

The cell cycle comprises a series of events during which a cell undergoes growth, genome duplication, and subsequent division. Aberrant cell cycle regulation leads to uncontrolled division and tumorigenesis. The precise temporal and spatial regulation of proteins is crucial for cell cycle progression. Numerous proteins exhibit cell cycle-dependent fluctuations in their abundance.^2^ However, deciphering the subcellular location of proteins across the cell cycle continues to pose a challenge. In the study by Litsios et al., they employed a high-content imaging approach with convolutional neural networks, allowing for the high-resolution mapping of proteome-level dynamics during the budding yeast cell cycle (Fig. 1).^1^

Litsios et al. implemented high-throughput imaging of cells expressing the proteins of interest tagged with GFP. Cells were automatically cleaved into single cells using a cell segmentation algorithm. By integrating neural network architectures, a category of deep learning algorithms frequently employed for analyzing image data, they developed a framework with CycleNET^1^ for classifying cell cycle stages of yeast cells, and DeepLoc^3^ for localizing proteins across 22 subcellular compartments. Using rigorous statistical scoring metrics, they identified 405 proteins (of approximately 3900) with periodic localization changes. Furthermore, they mapped proteome movements across cellular compartments and found that the primary sites where changes in protein localization occurred were the cytoplasm and the compartments linked to the bud and nucleus, while there were only minimal alterations observed in the mitochondria. To explore the periodicity of proteome concentration, they quantified the protein concentration using mean GFP pixel intensity. The global proteome concentration rose during the G1 phase, reached its highest point at the end of G1, and then dropped following the cell’s commitment to the cell cycle progression. Notably, the mitochondrial translational machinery exhibited distinct behavior compared to cytoplasmic translation because of the relatively stable concentration of mitochondrial proteins throughout the cell cycle. Litsios et al. identified 810 proteins with periodic concentrations. To elucidate the multilevel regulation of protein abundance periodicity, they performed RNA sequencing and ribosome profiling to measure transcription level and translation efficiency. Approximately 37% of the periodic proteome showed periodicity at either the transcript level or translational efficiency. Moreover, they characterized three typical dynamic patterns for genes that exhibit periodic changes at both the transcriptional and protein levels. The first two categories, which comprise the majority, either show simultaneous changes in mRNA and protein levels or a short lag where changes in protein levels occur after those in mRNA. The third category of genes, however, displays a significant delay between the changes in transcript levels and protein levels. Functional enrichment analysis of cell cycle periodic proteins indicated a specialization in their roles within the cell cycle, with proteins showing periodic concentration predominantly involved in cell cycle control and those with periodic localization associated with carrying out the cell cycle program. By mapping the protein dynamics throughout key cell cycle shifts onto flux networks, they revealed the coordinated movements of proteins. Intriguingly, they identified a previously uncharacterized protein, Ymr295c, which exhibits high periodicity in both concentration and distribution. With a localization profile similar to that of 40 other proteins, Ymr295c has been implicated in functions related to cell polarity and morphogenesis. The deletion of *YMR295C* further demonstrated that Ymr295c is involved in the regulation of cell wall synthesis. Protein-protein interaction assays identified interactions between Ymr295c and key enzymes involved in 1,3-beta-glucan synthesis. The study proposed the name GSR1 (Glucan Synthesis Regulator) for Ymr295c, recognizing it as a regulator of cell wall polysaccharide synthesis that was previously overlooked.

This study provides a valuable resource for understanding proteome changes during the cell cycle of the budding yeast *S. cerevisiae* and sets the stage for future studies. Future studies could explore the broader implications of these findings in other species, particularly in the human cell cycle, and how these periodic protein dynamics are related to disease states. Moreover, this study offers new insights into the functional analysis of uncharacterized genes with cell cycle-resolved phenomics. The integration of high-content imaging of live cells with convolutional neural network algorithms facilitates the efficient and precise analysis of large-scale microscopic data, providing a paradigm for spatiotemporal analyses of the proteome in eukaryotic systems. The web-based tool developed in this study will be beneficial for researchers exploring proteome dynamics in a user-friendly manner. This study investigated proteome dynamics using a yeast protein-GFP collection.^4^ However, GFP-tagged proteins may introduce biases, as not all proteins can be suitably tagged without affecting their native function or localization. Moreover, the budding yeast divides asymmetrically to produce a mother cell and a smaller daughter cell. The cell cycles of mother and daughter cells are very different. For example, the daughter cells spend more time growing in size before passing Start during G1, due to the sizer mechanisms. Since the images collected a mixture of both mother and daughter cells, whether this heterogeneity could affect the analysis results is also a question worth investigating. Furthermore, while this study focused on changes in protein abundance and localization throughout the cell cycle, it did not delve into the molecular mechanisms underlying these changes. For instance, post-translational modifications (PTMs) of proteins were not considered, which are crucial in regulating the cell cycle.^5^ Future studies could explore the functions of these PTMs during the cell cycle using advanced mass spectrometry analysis.

In summary, this study elucidated a high-resolution spatiotemporal map of proteome dynamics during the eukaryotic cell cycle, revealing the dynamic changes in proteins as they move and regulate the cell cycle progression. It also provides compelling evidence for investigating other species, particularly humans. This study sheds light on the effectiveness of integrating imaging, deep learning, and multi-omics data to dissect complex biological processes. It has the potential to drive research towards therapeutic breakthroughs in diseases characterized by cell cycle dysregulation.
